# AAV2-mediated GRP78 Transfer Alleviates Retinal Neuronal Injury by Downregulating ER Stress and Tau Oligomer Formation

**DOI:** 10.1167/iovs.18-24427

**Published:** 2018-09

**Authors:** Yonju Ha, Wei Liu, Hua Liu, Shuang Zhu, Fan Xia, Julia E. Gerson, Nisha A. Azhar, Ronald G. Tilton, Massoud Motamedi, Rakez Kayed, Wenbo Zhang

**Affiliations:** 1 Department of Ophthalmology and Visual Sciences, University of Texas Medical Branch, Galveston, Texas, United States; 2 Department of Ophthalmology, Union Hospital, Tongji Medical College, Huazhong University of Science and Technology, Wuhan, China; 3 Center for Biomedical Engineering, University of Texas Medical Branch, Galveston, Texas, United States; 4 Department of Neurology, University of Texas Medical Branch, Galveston, Texas, United States; 5 Internal Medicine, University of Texas Medical Branch, Galveston, Texas, United States; 6 Departments of Neuroscience, Cell Biology, and Anatomy, University of Texas Medical Branch, Galveston, Texas, United States

**Keywords:** traumatic optic neuropathy, retinal ganglion cell, endoplasmic reticulum stress, GRP78, adeno-associated virus, tau oligomers

## Abstract

**Purpose:**

Retinal ganglion cell (RGC) death following axonal injury occurring in traumatic optic neuropathy (TON) causes irreversible vision loss. GRP78 is a molecular chaperone that enhances protein folding and controls activation of endoplasmic reticulum (ER) stress pathways. This study determined whether adeno-associated virus (AAV)-mediated gene transfer of GRP78 protected RGCs from death in a mouse model of TON induced by optic nerve crush (ONC).

**Methods:**

ONC was induced by a transient crush of optic nerve behind the eye globe. AAV was used to deliver genes into retina. Molecules in the ER stress branches, tau oligomers, and RGC injury were determined by immunohistochemistry or Western blot.

**Results:**

Among tested AAV serotypes, AAV2 was the most efficient for delivering genes to RGCs. Intravitreal delivery of AAV2-GRP78 markedly attenuated ER stress and RGC death 3 days after ONC, and significantly improved RGC survival and function 7 days after ONC. Protein aggregation is increased during ER stress and aggregated proteins such as tau oligomers are key players in neurodegenerative diseases. AAV2-GRP78 alleviated ONC-induced increases in tau phosphorylation and oligomerization. Furthermore, tau oligomers directly induced RGC death, and blocking tau oligomers with tau oligomer monoclonal antibody (TOMA) attenuated ONC-induced RGC loss.

**Conclusion:**

These data indicate that the beneficial effect of AAV2-GRP78 is partially mediated by the reduction of misfolded tau, and provide compelling evidence that gene therapy with AAV2-GRP78 or immunotherapy with TOMA offers novel therapeutic approaches to alleviate RGC loss in TON.

Traumatic optic neuropathy (TON) is an acute injury of the optic nerve occurring during motor vehicle and bicycle accidents, falls, assaults, war, and natural disasters.^1^ To date, there is no effective therapy for treating TON, and retinal ganglion cell (RGC) death following optic nerve injury is a major cause of irreversible visual loss.^2–4^ Underlying pathophysiologic mechanisms are largely unknown.

The endoplasmic reticulum (ER) is an intracellular organelle for protein synthesis, folding, and trafficking. GRP78 (78-kDa glucose-regulated/binding immunoglobulin protein) is a molecular chaperone located in the ER that binds to PKR-like ER kinase (PERK), inositol-requiring enzyme 1 (IRE1), and activating transcription factor 6 (ATF6), keeping these three branches of the unfolded protein response (UPR) in an inactive state. When ER function is disturbed by various cellular stressors, protein folding is altered and ER stress is induced. As unfolded and misfolded proteins accumulate in the ER, the UPR is initiated and PERK, IRE1, and ATF6 are released from GRP78 to activate downstream signaling pathways. Prolonged or severe ER stress induces cell death and has been associated with numerous major diseases, including neurodegenerative diseases, stroke, diabetes, and cardiovascular diseases.^5–13^ Recently, it has been reported that ER stress is involved in RGC death in a mouse model of TON induced by optic nerve crush (ONC) in which C/EBP-homologous protein (CHOP) and XBP-1 differentially regulate RGC death.^10^ Nevertheless, underlying mechanisms of ER stress–induced RGC death remain largely unclear, and effective therapies to treat RGC injury in TON by targeting ER stress have not been developed.

Among ER stress pathways, GRP78, also known as BiP, is a master regulator by binding to the ER stress sensors to inhibit their activation and enhancing the ability of the ER to handle misfolded proteins to recover ER homeostasis.^5,14^ Consequently, boosting GRP78 expression represents a very effective way to prevent ER stress and protect cells from injury. In a rat model of autosomal dominant retinitis pigmentosa caused by the P23H mutation within the rhodopsin gene, overexpressing GRP78 alleviates ER stress and preserves photoreceptor function.^15^ Similarly, GRP78 overexpression reduces nigral dopamine loss and ameliorates behavioral deficits in a rat model of Parkinson's disease induced by elevated human α-synuclein.^14^ In the present study, we determined whether adeno-associated virus (AAV)2-mediated GRP78 overexpression protects RGC injury in TON and investigated potential mechanisms underlying the effects of GRP78.

## Methods

### Animals

Animal protocols were approved by the Institutional Animal Care and Use Committee of the University of Texas Medical Branch (Animal Protocol #1108033A). All experimental procedures and use of animals were performed in accordance with the Association for Research in Vision and Ophthalmology Statement for the Use of Animals in Ophthalmic and Vision Research. C57BL/6J mice were purchased from Jackson Laboratory (Bar Harbor, ME, USA) and maintained on a 12:12 light/dark cycle with food and water available ad libitum.

### Intravitreal Injection

Adeno-associated virus (AAV) carrying green fluorescent protein (GFP) cDNA driven by cytomegalovirus (CMV) early enhancer/chicken β actin (CAG) promoter (AAV[1, 2, 5, 6, 8, 9]-GFP) and self-complementary AAV9 (scAAV9) carrying GFP cDNA driven by CMV promoter were purchased from SignaGen Laboratories (Rockville, MD, USA). AAV2 carrying human GRP78 cDNA driven by CAG promoter was generated through the custom recombinant AAV (rAAV) vector production service of SignaGen Laboratories using plasmid pCMV GRP78-Myc-KDEL-wt (Addgene, Cambridge, MA, USA) as a template. Four-week-old mice were anesthetized by intraperitoneal injection of a mixture of ketamine hydrochloride (100 mg/kg) and xylazine hydrochloride (10 mg/kg) followed by intravitreal injection of 1 μL AAV1-GFP, AAV2-GFP, AAV5-GFP, AAV6-GFP, AAV8-GFP, AAV9-GFP, scAAV9-GFP, AAV2-GRP78, or AAV2-Null control (1 × 10^12^ vector genomes [VG]/mL) using a 35-gauge needle.^16^

### Retinal Imaging Using Confocal Scanning Laser Ophthalmoscopy (SLO)

Mice were anesthetized by intraperitoneal injection of a mixture of ketamine hydrochloride (100 mg/kg) and xylazine hydrochloride (10 mg/kg) at indicated time points (1, 2, 4, and 8 weeks) after intravitreal injection of AAV-GFP. Pupils were dilated using tropicamide and phenylephrine (Alcon, Fort Worth, TX, USA), and GenTeal Lubricant Eye Drops (Alcon) were used to prevent corneal drying. When fully anesthetized, mice were positioned on the imaging platform and all images were acquired in the high-resolution mode (512 × 512 pixels) over a 30° × 30° field of view with the Heidelberg Spectralis HRA system (Heidelberg Engineering, Franklin, MA, USA). Posterior pole of retinal fundus was chosen as a standard test area. Equivalent region of interest was captured at each time point in the infrared and fluorescence detection modes. The 488-nm laser was used for the excitation of green fluorescence, and a barrier filter at 500 nm was used to remove the reflected light with unchanged wavelength.^17^

### Induction of TON

ONC was used to induce TON as described.^17^ Briefly, 4 to 5 weeks after AAV2 injection, mice were anesthetized by intraperitoneal injection of ketamine hydrochloride (100 mg/kg) and xylazine hydrochloride (10 mg/kg); 0.5 % proparacaine was applied to the eye for local anesthesia before the procedure. After cutting conjunctiva around the eye globe, the optic nerve of right eye close to its origin in the optic disc was crushed for 10 seconds using forceps (Dumont RS5005; Roboz, Gaithersburg, MD, USA). The left eye served as control and was exposed to the same surgery without crushing. Eyes and retinas were collected for analysis at 3 and 7 days following the ONC procedures. To investigate the effect of tau oligomer monoclonal antibody (TOMA), mice were injected intravenously with TOMA (30 μg/mouse)^18^ or mouse IgG isotype control (Thermo Fisher Scientific, Waltham, MA, USA) 1 hour before the ONC procedure.

### Induction of Retinal Ischemia-Reperfusion

Mouse model of retinal ischemia-reperfusion was induced as described.^16^ Briefly, 4 to 5 weeks after AAV2 injection, mice were anesthetized and a 30-gauge infusion needle connected to a saline reservoir was inserted into the anterior chamber of the right eye. IOP was raised to 110 mm Hg for 45 minutes by elevating the saline reservoir. A sham procedure performed without elevating the pressure in the left eye served as the control. Eyes were collected for analysis at 7 days after transient ischemic injury.

### Immunostaining of Retinal Whole Mounts

Eyes were collected at 8 weeks after intravitreal injection of AAV-GFP or 7 days after TON, and fixed in 4% paraformaldehyde (PFA). Retinas were dissected from the choroid and sclera, blocked and permeabilized in PBS containing 5% normal goat serum and 0.3% Triton X-100 for 3 hours, and then incubated with Isolectin B4 *(Griffonia simplicifolia)* (1:200; Life Technologies, Rockville, MD, USA), or antibodies against Tuj-1 (1:400; BioLegend, San Diego, CA, USA), glial fibrillary acidic protein (GFAP) (1:500; Dako, Carpinteria, CA, USA) or Iba-1 (1:400; Wako, Osaka, Japan) overnight at 4°C. Subsequently, retinas were incubated with Alexa Fluor 594-conjugated goat anti-mouse or goat anti-rabbit secondary antibodies (1:400; Life Technologies). After washing with PBS, retinas were mounted on slides using Vectashield mounting medium (Vector Laboratories, Burlingame, CA, USA), and representative images were taken by confocal (LSM 510 Meta; Carl Zeiss Inc, Thornwood, NY, USA) or epifluorescence (Olympus, Waltham, MA, USA) microscopy.

### Immunostaining of Retinal Frozen Section

Eyes were fixed with 4% PFA in 0.1 M phosphate buffer for 60 minutes on ice. Then eyes were immersed in 30% sucrose solutions in PBS overnight, embedded in optimum cutting temperature medium, and cut into 10-μm-thick sections for immunofluorescence staining. Retinal frozen sections were post-fixed with 4% PFA in PBS for 10 minutes, rinsed with PBS, permeabilized with PBS containing 0.1% Triton X-100 for 15 minutes at room temperature, and blocked with PowerBlock (Biogenx, San Ramon, CA, USA) for 1 hour. Subsequently, sections were incubated with primary antibodies against GRP78 (1:500), p-PERK (1:300; Cell Signaling Technology, Beverly, MA, USA), ATF6 (1:250; Origene Technologies Inc., Rockville, MD, USA), ATF4 (1:150; Cell Signaling), CHOP (1:200; Santa Cruz Biotechnology, Santa Cruz, CA, USA), T22 (1:500),^19^ AT180 (1:500; Thermo Fisher Scientific), and Alexa Fluor 647-conjugated Calreticulin (1:400; Abcam, Cambridge, MA, USA). After rinsing, retinal sections were incubated with appropriate Alexa Fluor 488- or 594-labeled secondary antibodies at room temperature for 1 hour, mounted with Fluoroshield with 4′,6-diamidino-2-phenylindole (DAPI) histology mounting medium (Sigma-Aldrich, St. Louis, MO, USA), and mid-central region of each retinal section was imaged with epifluorescence microscopy or confocal microscopy. Fluorescence intensities from ganglion cell layer (GCL), inner nuclear layer (INL), and outer nuclear layer (ONL) were measured and normalized to area using ImageJ software (http://imagej.nih.gov/ij/; provided in the public domain by the National Institutes of Health, Bethesda, MD, USA). Of note, to avoid quantization errors when using immunostaining method, retinal sections from all experimental groups were stained with the antibody against that specific molecule in parallel; images of all experimental groups were taken by the same fluorescent microscopy with the same parameters, including excitation fluorescent intensity, exposure time, gain, brightness and contrast; and brightness and contrast of images of experimental groups were adjusted simultaneously during image processing.

### Western Blotting

Retinas were collected 4 weeks after intravitreal injection of AAV2-GRP78 or AAV2-Null. Proteins were extracted in RIPA buffer (50 mM Tris-HCl pH 7.4, 150 mM NaCl, 0.25% deoxycholic acid, 1% NP-40, 1 mM EDTA) containing protease inhibitors, separated on 10% SDS-PAGE gels and electroblotted onto polyvinylidene difluoride membranes that were incubated with primary antibody for GRP78 (BD Biosciences, San Jose, CA, USA). α-Tubulin was probed with a mouse monoclonal anti-α-Tubulin (Sigma-Aldrich) as a loading control. Proteins were detected using the enhanced chemiluminescence (ECL) system (Pierce, Rockford, IL, USA) and protein expression was quantified using ImageJ.

### Isolation of Primary RGCs

Primary RGCs were isolated from wild-type (WT) mouse pups at postnatal day 4 to 5 as described previously.^17^ Briefly, collected retinas were dissociated in a papain solution (15 U/mL) at 37°C for 15 minutes, and macrophages and microglial cells were removed by incubation with anti-macrophage antiserum. Nonadherent cells were incubated with mouse Thy-1.2 antibody (BD Biosciences) to isolate ganglion cells. Cells were seeded at a density of 2.3 × 10^5^ cells per well and treated with tau oligomers (100 ng/mL).^20^ At 24 hours after treatment, cells were exposed to the TUNEL assay to detect cell death.

### TUNEL Assay

To visualize apoptotic cells, TUNEL assay was performed on retinal frozen sections or primary RGCs with ApopTag Fluorescein In Situ Apoptosis Detection Kit (EMD Millipore, Billerica, MA, USA) according to the manufacturer's instructions. Retinal sections or RGCs were counterstained with DAPI to label nuclei; images were taken using epifluorescence microscopy; and TUNEL-positive cells were counted. The percentage of apoptotic RGCs in vitro was calculated as the ratio of TUNEL-positive cells to DAPI-positive cells per field of view.

### Dark-Adapted ERG Analysis

ERG analysis was performed 7 days after ONC as described previously.^17^ Briefly, mice were dark-adapted overnight, anesthetized by intraperitoneal injection of a mixture of ketamine hydrochloride (100 mg/kg) and xylazine hydrochloride (10 mg/kg). Following pupil dilation with a mixture of atropine and phenylephrine, gold ring electrodes were placed on the center of the cornea and ERG recordings were obtained using the Espion system (Diagnosys LLC, Lowell, MA, USA). Brief white flashes were presented from dim to bright with the interstimulus interval increasing with brightness. Positive scotopic threshold responses (pSTRs) were recorded in response to briefly flashed stimuli of −4.3 to −3.2 log cd-s/m^2^. pSTRs were measured 110 ms after the flash onset. Each record was an average of at least 50 responses.

### High-Resolution En Face Optical Coherence Tomography (OCT)

Spectral-domain (SD)-OCT was used to noninvasively analyze retinal neuronal structure.^21^ Mice were anesthetized with an intraperitoneal injection of a mixture of ketamine (100 mg/kg) and xylazine (10 mg/kg). Pupils were dilated with tropicamide and phenylephrine, and mice were then positioned on the imaging platform of the Spectral-Domain Ophthalmic Imaging System (Envisu R2200; Bioptigen Inc., Morrisville, NC, USA). An en face scan consisting of 100 B-scans in a rectangular pattern was performed, in which high-resolution B-scans consist of 1000 A-scans with 1.4-μm resolution and 1.4-mm width. Retinal thickness within a donut-shaped area centered at the optic nerve disc was measured in an annular pattern. The inner and outer radiuses of the donut-shaped area were 200 and 700 μm from the center of the optic nerve disc, respectively. Retinal thickness maps and statistics were obtained using Bioptigen's report generator. The analysis included the “mouse report” template, which uses techniques for automated segmentation of the retinal images to generate depth maps and statistical tables of each retinal layer at various depths. The ganglion cell complex (GCC) is defined as the three innermost retinal layers: the nerve fiber layer, the GCL, and the inner plexiform layer.

### Statistical Analysis

Data were presented as mean ± SEM and analyzed by Student's *t*-test or 1-way ANOVA followed by Newman Keuls post hoc test. Statistical analysis was conducted using GraphPad Prism program (GraphPad Software Inc., La Jolla, CA, USA). A *P* value < 0.05 was considered statistically significant.

## Results

### AAV2 Efficiently Delivers GRP78 Into RGCs

**Figure 1:**
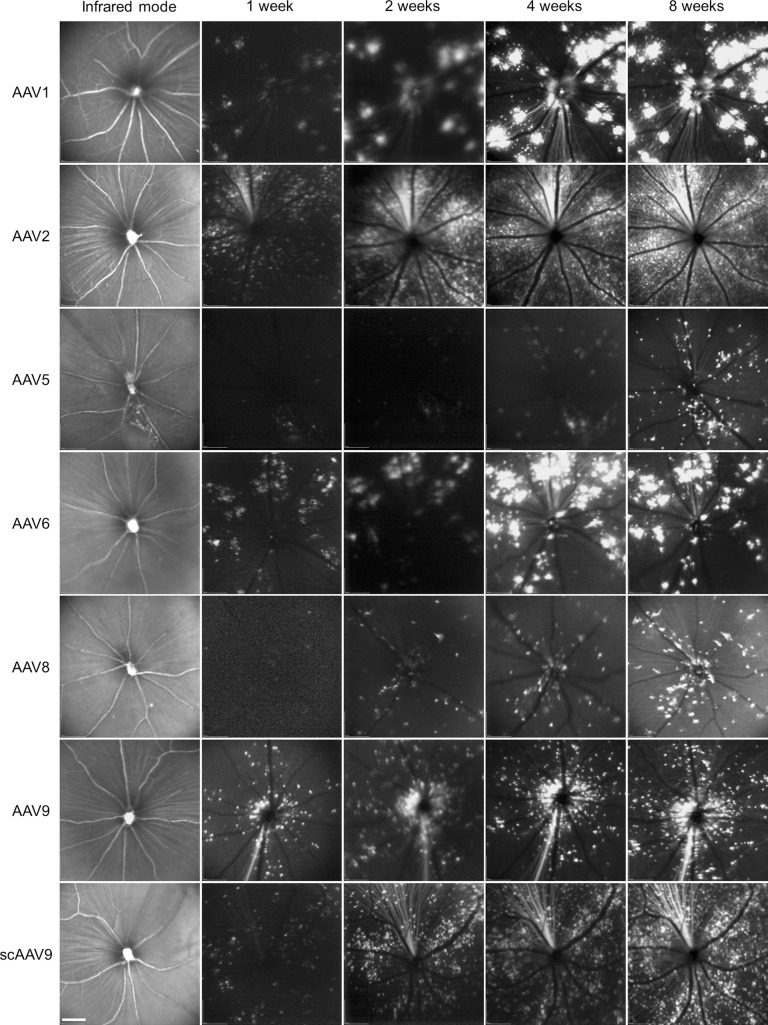
Transduction profiles of different AAV serotypes encoding GFP in the retina. Various AAV serotypes (AAV1, 2, 5, 6, 8, 9) and scAAV9 containing a GFP reporter were intravitreally injected into the eyes of 4-week-old mice. Representative fundus images of the retina were taken by SLO in the fluorescence detection mode at 1, 2, 4, and 8 weeks after injection. Preliminary fundus images were captured in the infrared mode prior to injection. Scale bar: 200 μm.

AAV is a nonpathogenic human parvovirus that is considered a promising gene therapy vehicle due to long-term transgene expression and relative lack of immune response and toxicity.^22^ To determine which AAV serotype can deliver genes into RGCs with high efficiency, we intravitreally injected various AAV serotypes carrying GFP into the eyes of 4-week-old mice and examined GFP expression in the retinas at increasing time intervals after injection by noninvasive SLO (Fig. 1). During an 8-week follow-up, GFP-positive cells were detected in the retinas at 1 week after injection and progressively increased over time. At 4 weeks after injection, significant GFP expression was observed in the retinas, although marked variation in expression level and distribution was observed among the seven tested AAV serotypes. AAV2 delivered GFP into superficial retinal cells with the highest transduction efficiency and uniformity.

**Figure 2:**
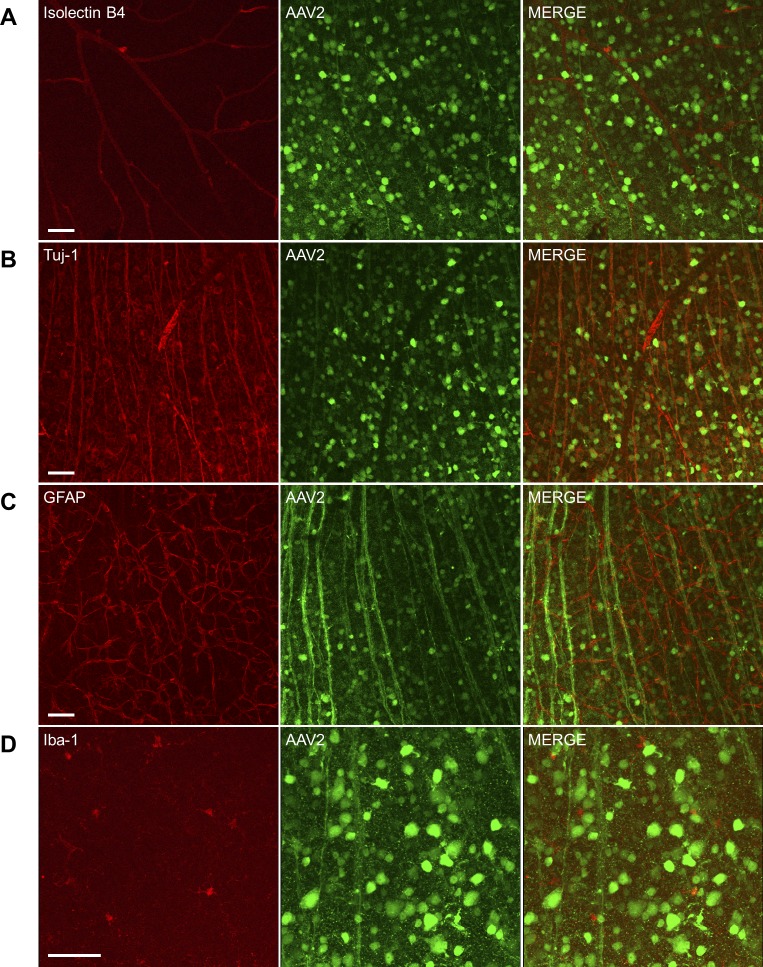
Transduction characteristics of AAV2-GFP in the retina. Mice received intravitreal injection of AAV2-GFP (1 μL, 1 × 10^12^ vector genomes/mL) in both eyes. Eight weeks after injection, retinas were collected and stained with Isolectin B4 (A, red, vessels), anti-Tuj-1 (B, red, RGCs), anti-GFAP (C, red, astrocytes), and anti-Iba-1 (D, red, microglia/monocytes). Confocal images were captured using ×20 (A–C) or ×40 (D) objective. Scale bar: 50 μm.

To further determine cell types infected with each AAV serotype, we collected retinas 8 weeks after intravitreal AAV injection, and then stained with Isolectin B4 (vasculature) or antibodies against Tuj-1 (RGCs), GFAP (glia), and Iba-1 (microglia/monocytes). GFP-positive RGCs (GFP^+^/Tuj-1^+^) were observed by confocal microscopy regardless of AAV serotypes (Fig. 2, Supplementary Figs. S1–S6). Consistent with SLO analysis, AAV2 delivered GFP into both RGC somas and axons with the highest efficiency (Fig. 2). In contrast, AAV1, AAV5, AAV6, AAV8, and AAV9 delivered GFP into only a small percentage of retinal cells (Supplementary Figs. S1–S5). Although scAAV9 also delivered GFP into a significant number of RGCs (Supplementary Fig. S6), its specificity and efficiency to RGCs were relatively low and less GFP-positive axons were observed versus AAV2. Moreover, scAAV9 can only encapsulate DNA less than 1 kb in length, which excludes it as a vector of choice to deliver GRP78 cDNA (approximately 2 kb) into retinal cells. In summary, these data demonstrated that AAV2 was the best vehicle for introducing GRP78 into RGCs.

**Figure 3:**
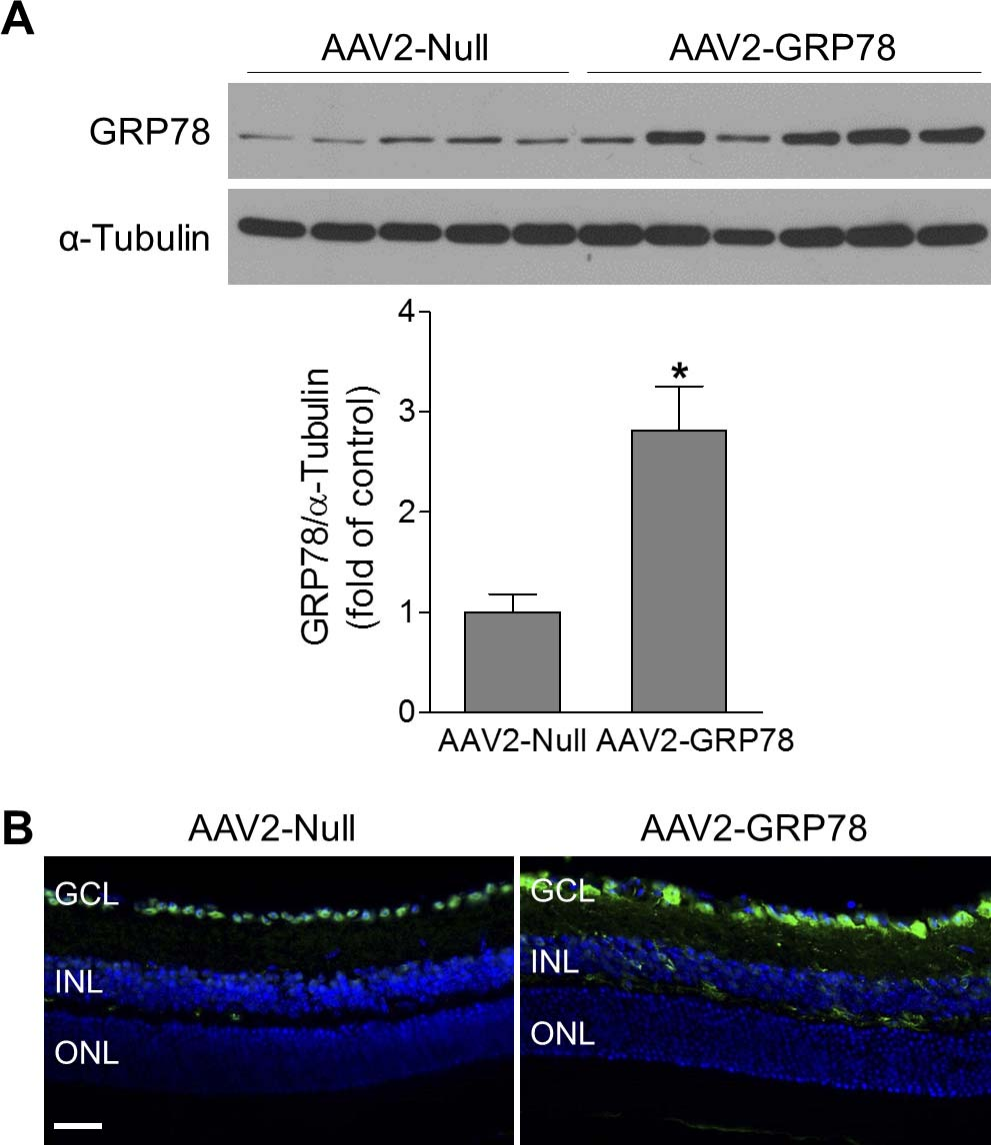
GRP78 is specifically delivered into RGCs. Retinas or eyeballs were collected at 4 to 5 weeks after intravitreal injection of AAV2-Null or AAV2-GRP78. (A) The expression level of GRP78 was evaluated by Western blotting. α-Tubulin was used as a loading control. Bar graph represents quantification of retinal GRP78 expression. n = 5–6. *P < 0.05 versus AAV2-Null. (B) Representative images of GRP78 immunostaining (green) in retinal frozen sections. DAPI: blue, for nuclei. n = 4. Scale bar: 50 μm.

We therefore generated AAV2 carrying human GRP78 cDNA under the control of CMV early enhancer/chicken β actin (CAG) promoter and examined AAV2-mediated delivery of GRP78 into retinal cells. Four weeks after intravitreal injection of AAV2, GRP78 protein expression was significantly increased (approximately 2.8-fold) in retinas of mice receiving AAV2-GRP78 versus those receiving AAV2-Null (Fig. 3A). The distribution of GRP78 in the retina was further analyzed by immunostaining. Similarly, GRP78 immunoreactivity was markedly increased and mainly localized in neurons in the GCL, although some cells in the INL were also stained positive for GRP78 (Fig. 3B).

### AAV2-GRP78 Attenuates ER Stress and Prevents RGC Injury

**Figure 4:**
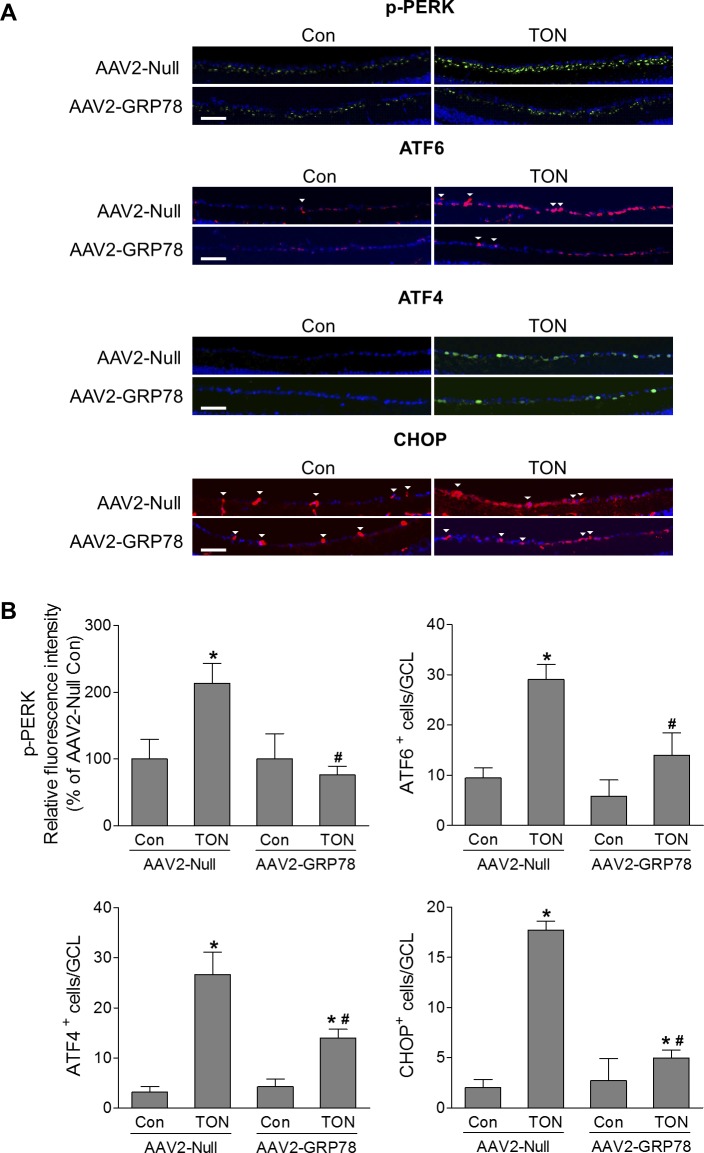
GRP78 attenuates TON-induced ER stress. TON was performed at 4 to 5 weeks after intravitreal injection of AAV2-Null or GRP78, and eyes were collected at 3 days after TON. Immunostaining was performed on retinal frozen sections. (A) Representative images of p-PERK, ATF6, ATF4, and CHOP expressions in the GCL. Arrowheads indicate nonspecific vessel staining. (B) Bar graphs represent quantification of p-PERK, ATF6, ATF4, and CHOP. n = 3–4, *P < 0.05 versus relevant control, #P < 0.05, AAV2-Null-TON versus AAV2-GRP78-TON. Scale bar: 50 μm.

To further investigate whether AAV2-mediated GRP78 overexpression is neuroprotective in retinal diseases, we used an ONC mouse model that has been widely used to study mechanisms and strategies for neuroprotection in TON.^17^ In this model, optic nerve injury is induced by a transient crush that triggers a degenerative response spreading retrogradely to RGC soma. Three days after TON, analysis of ER stress–related molecules by immunostaining revealed marked increases in p-PERK, ATF6, ATF4, and CHOP in GCL cells from retinas infected with AAV2-Null, indicating ER stress was induced after optic nerve injury. In contrast, in TON retinas infected with AAV2-GRP78, immunoreactivity of p-PERK was reduced to preinjury level and the number of ATF6, ATF4, and CHOP-positive GCL cells was significantly reduced by 52%, 47%, and 72%, respectively (Fig. 4). Immunoreactivities of p-PERK, ATF6, ATF4 and CHOP were not changed in the INL (Supplementary Fig. S7) and not detected in the ONL (data not shown). These data indicate that GRP78 overexpression mitigates ER stress in RGCs during TON.

**Figure 5:**
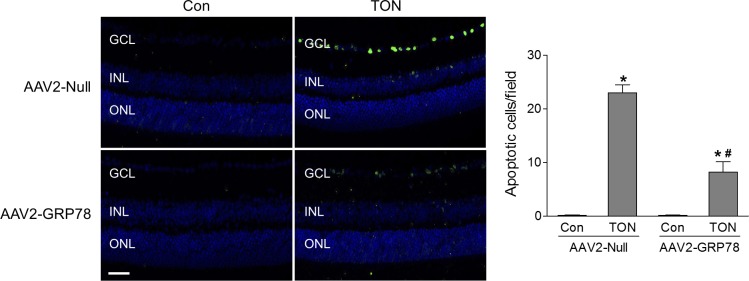
GRP78 overexpression attenuates RGC death after TON. TON was performed at 4 to 5 weeks after intravitreal injection of AAV2-Null or GRP78 and retinas were collected at 3 days after TON. TUNEL assay was performed on retinal frozen sections. Green fluorescence reflects TUNEL-positive cells and blue fluorescence was DAPI staining of cell nuclei. Bar graph represents the number of apoptotic RGCs per field of view. n = 3–4, *P < 0.05 versus relevant control, #P < 0.05 AAV2-Null-TON versus AAV2-GRP78-TON.

**Figure 6:**
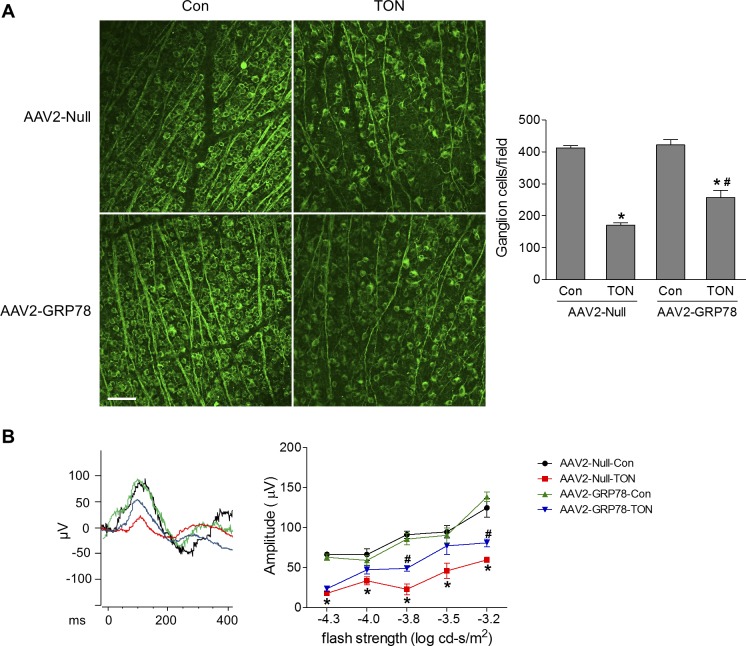
Overexpression of GRP78 prevents the cellular/functional loss of RGCs after TON. TON was performed at 4 to 5 weeks after intravitreal injection of AAV2-Null or GRP78. At 7 days after TON, mice were subjected to ERG followed by collection of the eyes. (A) Representative images of retinal flatmounts labeled with Tuj-1 antibody (green). Bar graph represents the number of Tuj-1–positive cells per field. Scale bar: 50 μm. (B) Left: Representative pattern of pSTR to a stimulus of −3.8 log cd-s/m^2^; Right: Average amplitudes of pSTR over a range of stimulus strengths. n = 5–6, *P < 0.05 versus respective control, #P < 0.05 AAV2-Null-TON versus AAV2-GRP78-TON.

Associated with activation of ER stress, TUNEL assay revealed that RGC death was significantly increased in AAV2-Null–infected retinas 3 days after TON, whereas this increase was significantly attenuated in AAV2-GRP78–infected retinas (Fig. 5). At 7 days after TON, the density of RGCs was determined in Tuj-1–stained retinal flatmounts. We found RGCs were reduced by 59% after TON, which was partially prevented by GRP78 overexpression (Fig. 6A). To assess the functional consequence of AAV2-GRP78 delivery, ERG was performed to examine RGC function at 7 days after TON. The scotopic threshold responses (STRs) are the most sensitive responses in the dark-adapted ERG,^23^ and the positive STR (pSTR) is mainly generated by RGCs in the mouse retina and serves as a useful measurement of RGC electrical function.^17,24,25^ As shown in Figure 6B, the amplitudes of pSTRs in response to different flash strengths were significantly reduced in AAV2-Null–infected retinas after TON, indicating RGC dysfunction. However, this reduction in pSTR amplitudes was significantly attenuated in retinas infected with AAV2-GRP78. To test whether GRP78 gene delivery was neuroprotective under other injury conditions in addition to TON, we examined RGC loss in a mouse model of retinal ischemia-reperfusion induced by transient elevation of IOP.^16^ Similarly, we found that AAV2-GRP78 significantly prevented RGC loss after ischemic injury (Supplementary Fig. S8). Taken together, these data indicate that reduction of ER stress by AAV2-GRP78 provides functional neuroprotection for RGC during retinal neuropathy.

### GRP78 May Protect RGCs by Preventing Formation of Misfolded Tau After TON

**Figure 7:**
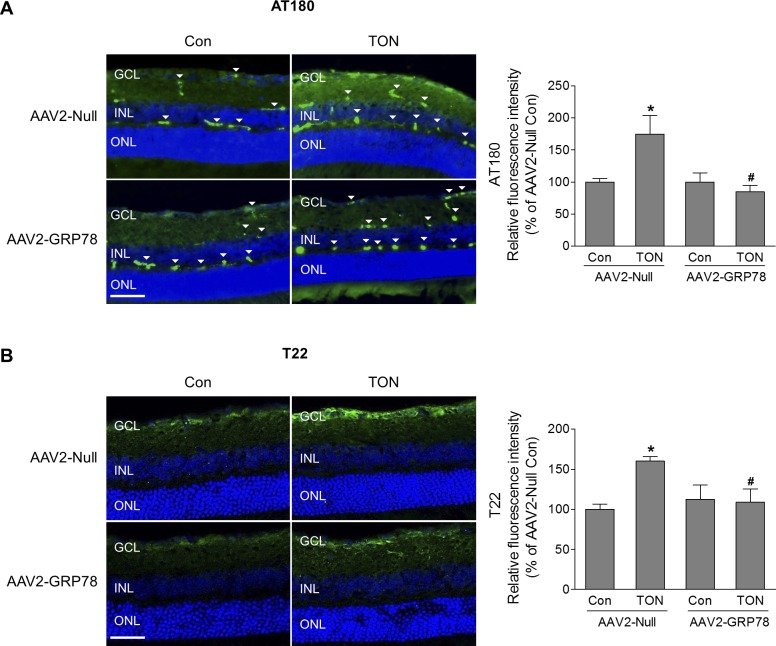
GRP78 overexpression decreases TON-induced tau phosphorylation and oligomerization. At 4 to 5 weeks after intravitreal injection of AAV2-Null or GRP78, mice were subjected to TON. Eyes were collected for frozen sections 3 days after TON. Representative images of phosphorylated tau (A) and tau oligomer (B) (green) are shown. Arrowheads indicate nonspecific vessel staining. Bar graphs represent quantification of immunoreactivity of phosphorylated tau and tau oligomer. n = 3–4. *P < 0.05 AAV2-Null-Con versus AAV2-Null-TON, #P < 0.05 AAV2-Null-TON versus AAV2-GRP78-TON. Scale bar: 50 μm.

Tau is a soluble microtubule-associated protein that is important for the maintenance and function of axons by binding to and stabilizing microtubules.^26^ In pathological conditions, modifications of tau by hyperphosphorylation, oxidation, mutation, and truncation can lead to formation of misfolded and aggregated tau, which plays a critical role in many neurodegenerative diseases in the central nervous system (CNS).^26^ Although neurofibrillary tangles (NFTs) formed by deposition of aggregated tau are a hallmark of Alzheimer's disease, increasing evidence indicates that tau oligomers (soluble and intermediate tau aggregates) but not NFTs are the main toxic tau species in Alzheimer's disease, traumatic brain injury, and other neurodegenerative tauopathies.^26,27^ Because disturbance of ER homeostasis leads to misfolding and aggregation of disease-related proteins, we investigated tau phosphorylation at Thr231, which is critical for tau's hyperphosphorylation^28–30^ by AT180 monoclonal antibody and examined the level of tau oligomers by anti-tau oligomer antibody T22, together with antibody against Calreticulin (ER marker) after TON. We found there was significant colocalization of phosphorylated tau or tau oligomer with Calreticulin (Supplementary Fig. S9), indicating accumulation of phosphorylated tau and tau oligomer in the ER during TON. Meanwhile, a portion of phosphorylated tau and tau oligomer were localized outside of the ER (Supplementary Fig. S9). Because GRP78 can enhance the ability of ER to handle misfolded proteins, we further tested if introduction of AAV2-GRP78 could reduce formation of misfolded tau. At 3 days after TON, significant increase in tau phosphorylation in the GCL was observed in mice infected with intravitreal AAV2-Null after TON. However, in retinas infected with AAV2-GRP78, TON-induced tau phosphorylation was remarkably reduced (Fig. 7A). Correlated with the alteration of tau phosphorylation, the level of tau oligomers was significantly increased in TON, and was blocked by introduction of GRP78 to the retina (Fig. 7B). Immunoreactivities of phosphorylated tau and tau oligomers, however, were not changed in the INL and the ONL (Supplementary Fig. S10).

**Figure 8:**
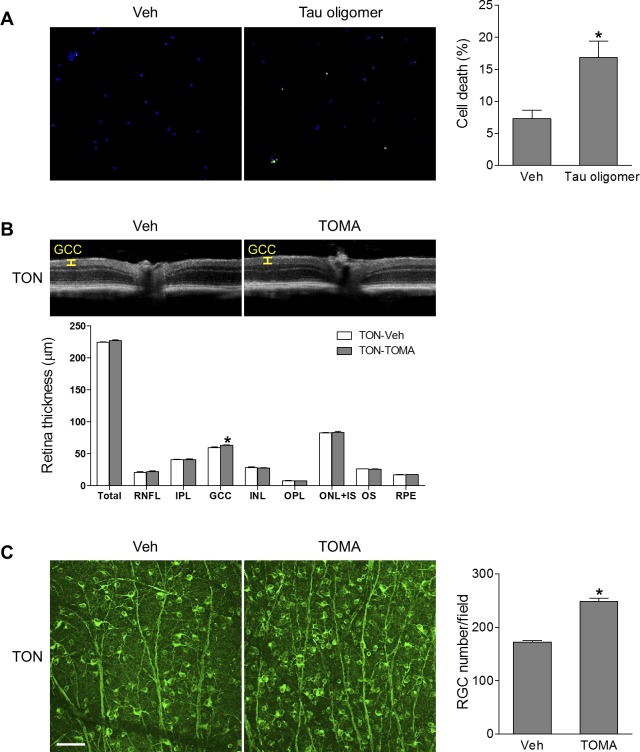
Tau oligomers are involved in RGC death. (A) Isolated primary mouse RGCs were exposed to Tau oligomers (100 ng/mL) or vehicle (Veh) for 24 hours followed by TUNEL assay. Green fluorescence reflects TUNEL-positive cells and blue fluorescence (DAPI) labels nuclei. Bar graph represents the percentage of apoptotic RGCs, calculated as the ratio of the number of TUNEL-positive cells to the total number of DAPI-stained nuclei. Eighteen fields were counted in each group; n = 3 independent experiments. *P < 0.05 versus Veh. (B, C) WT mice were intravenously injected with vehicle (IgG) or TOMA (30 μg/mouse) 1 hour before TON. (B) OCT analysis was conducted for retinal thickness 7 days after TON, and representative images are shown. Yellow lines indicate the GCC (composed of the RNFL, GCL, and IPL). Bar graph represents the thickness of total retina, individual retinal layers, and the GCC. IPL, inner plexiform layer; IS, inner segment; OPL, outer plexiform layer; OS, outer segment; RNFL, retinal nerve fiber layer. (C) Images of retinal flatmounts labeled with anti-Tuj-1 antibody (green). Bar graph represents the number of Tuj-1–positive cells per field. n = 6, *P < 0.05 versus Veh. Scale bar: 50 μm.

To determine whether tau oligomers can induce RGC injury, we purified and cultured primary mouse RGCs (Supplementary Fig. S11) and treated cells with tau oligomers for 24 hours. We found RGC death, as determined by TUNEL assay, was significantly increased by tau oligomer treatment, indicating tau oligomers are toxic to RGCs (Fig. 8A). Studies have shown that intravenous delivery of TOMA neutralized extracellular tau oligomers and promoted the egress of intracellular oligomeric tau, resulting in a net decrease of tau oligomers in the CNS and eventual serum clearance, and reversed the neurodegenerative phenotypes in aged P301L tau transgenic mice.^18,31,32^ To test whether tau oligomers were involved in RGC injury in TON, we intravenously injected TOMA to mice at 1 hour before ONC, and at 7 days after ONC, we used noninvasive OCT to assess the thickness of total retina, individual layers, and the GCC. The GCC is composed of the three innermost retinal layers: the nerve fiber layer, the GCL, and the inner plexiform layer; and the thinning of the GCC correlates well with the loss of RGCs in the microbeads occlusion model.^33^ We observed that TOMA treatment significantly preserved the thickness of the GCC layer compared with control IgG-treatment (Fig. 8B). Consistently, RGC loss examined by anti-Tuj1 immunostaining was reduced after TOMA treatment (Fig. 8C). Combination of both TOMA and AAV2-GRP78 therapies, however, did not further enhance the neuroprotective effect of AAV2-GRP78 (Supplementary Fig. S12). Taken together, these results suggest that introduction of GRP78 protects RGCs from injury in TON in a process involving inhibition of the formation of misfolded tau, and pharmacological blockade of tau oligomers represents a promising approach to alleviate RGC injury in TON.

## Discussion

As TON is critically involved in vision loss after accidents or assaults, but no proven therapy is available, interventions that can save and reverse retinal function are highly desired.^17^ Here we found that GRP78, a key controller of the ER stress pathway, represents a promising target to treat TON. Our data show that boosting GRP78 alleviates ER stress, reduces misfolded tau, protects RGC from death, and rescues RGC function, which is in line with other studies showing GRP78 overexpression is beneficial in autosomal dominant retinitis pigmentosa and Parkinson's disease.^14,15^ This study, together with previous studies that modulations of other molecules in ER stress branches, such as blocking CHOP and eukaryotic translation initiation factor 2α (eIF2α) or overexpressing Xbp-1s, also protect axonal injury-induced RGC death,^10,34^ highlights the potential value of regulation of ER stress for TON therapy. Indeed, accumulating evidence indicates that ER stress appears as a common mechanism for retinal neuropathies.^10,15,35^ In a mouse model of optic neuritis, RGC loss is prevented by simultaneously blocking eIF2α while activating Xbp-1s by AAV2-mediated gene delivery either before or at 3 days after induction of the injury.^35^ In our study, as it took at least 2 weeks to establish prominent GRP78 expression in RGCs after virus infection while RGCs underwent apoptosis shortly (in 3 days) after optic nerve crush, it leaves no window to assess the therapeutic effects of delivery of AAV2-GRP78 after ONC. However, under clinical settings, AAV2-GRP78 may offer an approach to protect RGCs after traumatic brain injury, because many patients do not develop vision loss until months after injury. Alternatively, chemical compounds that can immediately, potently, and sustainably induce GRP78 would be valuable tools to treat TON.

Many intracellular signaling pathways are involved in ER stress–induced cell death. PERK phosphorylates and inactivates eIF2α, leading to attenuation of global mRNA translation in order to reduce protein load in the ER and reestablish ER homeostasis. Meanwhile, phosphorylation of eIF2α triggers preferable translation of selected genes, such as ATF4, which causes upregulation of CHOP, leading to cell apoptosis by CHOP-mediated downregulation of anti-apoptotic proteins and upregulation of proapoptotic proteins in the BCL2 family, increases of reactive oxygen species, and suppression of cell cycle regulator protein 21.^36^ Calcium ions, released from the ER lumen during ER stress, can also induce cell death by activating caspase-12 via calcium-dependent protease calpain and by activating calcium/calmodulin-dependent protein kinase II.^36^ In our study, AAV2-GRP78–mediated neuroprotection may involve its direct regulation of proapoptotic signaling in the ER stress pathways, as intravitreal delivery of AAV2-GRP78 attenuates TON-induced CHOP expression. In addition to the direct effects, our data suggest that the neuroprotective effect of AAV2-GRP78 involves its regulation of formation of misfolded tau, a microtubule-associated protein. We demonstrated that levels of phosphorylated tau and tau oligomers were increased in TON and AAV2-GRP78 blocked such increases. We also found that tau oligomers were toxic to RGCs and critically involved in RGC injury in TON. Although the pathological aggregation of tau and its subsequent deposition in NFTs are defining histopathological features of Alzheimer's disease and many other neurodegenerative disorders in the CNS, such as progressive supranuclear palsy and traumatic injury,^27,37–39^ the role of tau in retinopathy is largely unknown. Only a few studies reveal increases in tau phosphorylation in the retinas of mice expressing mutant tau protein and in a rat model of glaucoma.^40–42^ Our study represents the first study in the field of retinopathy that not only showed tau phosphorylation and aggregation in a model of retinopathy, but also demonstrated that misfolded tau contributed to retinal neuronal injury. TOMA, which has been used to alleviate neurodegeneration in tauopathies,^18,43,44^ may provide a novel therapeutic approach to save retinal neurons in TON as well as other retinal diseases involving aggregated tau.

Mechanisms by which GRP78 regulates tau phosphorylation and aggregation in TON remain to be elucidated. GRP78, as a molecular chaperone, binds to unfolded or misfolded proteins to facilitate de novo protein folding and assembly, and targets misfolded proteins for degradation via ER-associated protein degradation or autophagy.^45^ These functions help to prevent formation and accumulation of tau oligomers in the ER. Because tau oligomers can propagate and be secreted,^20^ reduction of tau oligomers in the ER will also decrease tau oligomers in the cytosol and in the extracellular space. Moreover, boosting GRP78 increases the capacity of the ER to handle unfolded and misfolded proteins, and therefore reduces the amount of unfolded and misfolded proteins exiting from the ER to the proteasome for degradation. As a result, the proteasome can have more capacity to degrade misfolded cytosolic proteins, including tau oligomers. Similar to our findings, GRP78 has been found to interact with the misfolded form of the prion protein and inhibit its accumulation and propagation, and play a key role in the defense against prion diseases.^46^ In addition to direct regulation of proper tau protein folding, GRP78 may inhibit tau phosphorylation and aggregation by preventing PERK activation. PERK is known to activate GSK3β, which is one of the key kinases for tau phosphorylation, activate caspases that cleave tau, and directly phosphorylate tau on residues.^47^ These features have positioned PERK activation as an inducer for tauopathy and neurodegeneration, as tau phosphorylation and cleavage favor tau aggregation. Although future studies are needed to test these possibilities to elucidate the link between GRP78 and tau aggregation, boosting GRP78 expression may offer a promising approach to relieve tauopathy in the retina during TON as well as other neurodegenerative diseases in the brain.

Of note, multiple versions of ONC have been used in different laboratories, ranging from short time (3 seconds)^48,49^ and intermediate time (10 seconds),^50–54^ to long time (30–60 seconds).^55,56^ In our study, we used the 10-second crush protocol developed by Templeton and Geisert,^52^ in which it is easier to control crush time than in the 3-second crush protocol. This protocol produces consistent RGC loss^52^ and has been widely used.^50–54^ Although RGC death in the 10-second crush protocol is induced more rapidly and severely than in the 3-second crush protocol, RGC death in this model was caused by the damage of the axons within the optic nerve but not by alterations in blood flow to the retina.^52^ Moreover, retina is resistant to ischemia. Induction of retinal ischemia by blocking central retinal artery for 30 minutes followed by reperfusion did not cause any histological changes in the retina.^57^ Therefore, it is unlikely that crushing the optic nerve for 10 seconds will cause RGC death due to ischemia. This notion was supported by our data showing only RGCs underwent apoptosis after crush (Fig. 5), which is in contrast to neuronal apoptosis occurring in both GCL and INL after ischemic injury.^16,58^ Overall, RGC death in the 10-second crush protocol was caused by axonal injury rather than retinal ischemia.

In summary, here we demonstrated that AAV2-GRP78 protects RGC injury after TON via attenuating ER stress as well as preventing accumulation of tau oligomers. As ER stress is involved in many retinopathies, including diabetic retinopathy, glaucoma, and AMD, our study warrants further investigation of the ER stress/tauopathy pathways in these diseases and evaluation of whether boosting GRP78 expression and blocking tau oligomers are also beneficial in these diseases.

## Supplementary Material

Supplement 1
