# Supplementary material for: AAV2-mediated GRP78 Transfer Alleviates Retinal Neuronal Injury by Downregulating ER Stress and Tau Oligomer Formation
Source: Invest Ophthalmol Vis Sci. 2018 Sep;59(11):4670–82. doi: 10.1167/iovs.18-24427 (PMC6155472; doi:10.1167/iovs.18-24427)
Supplement: Supplement 1 [file iovs-59-11-21_s01.pdf]

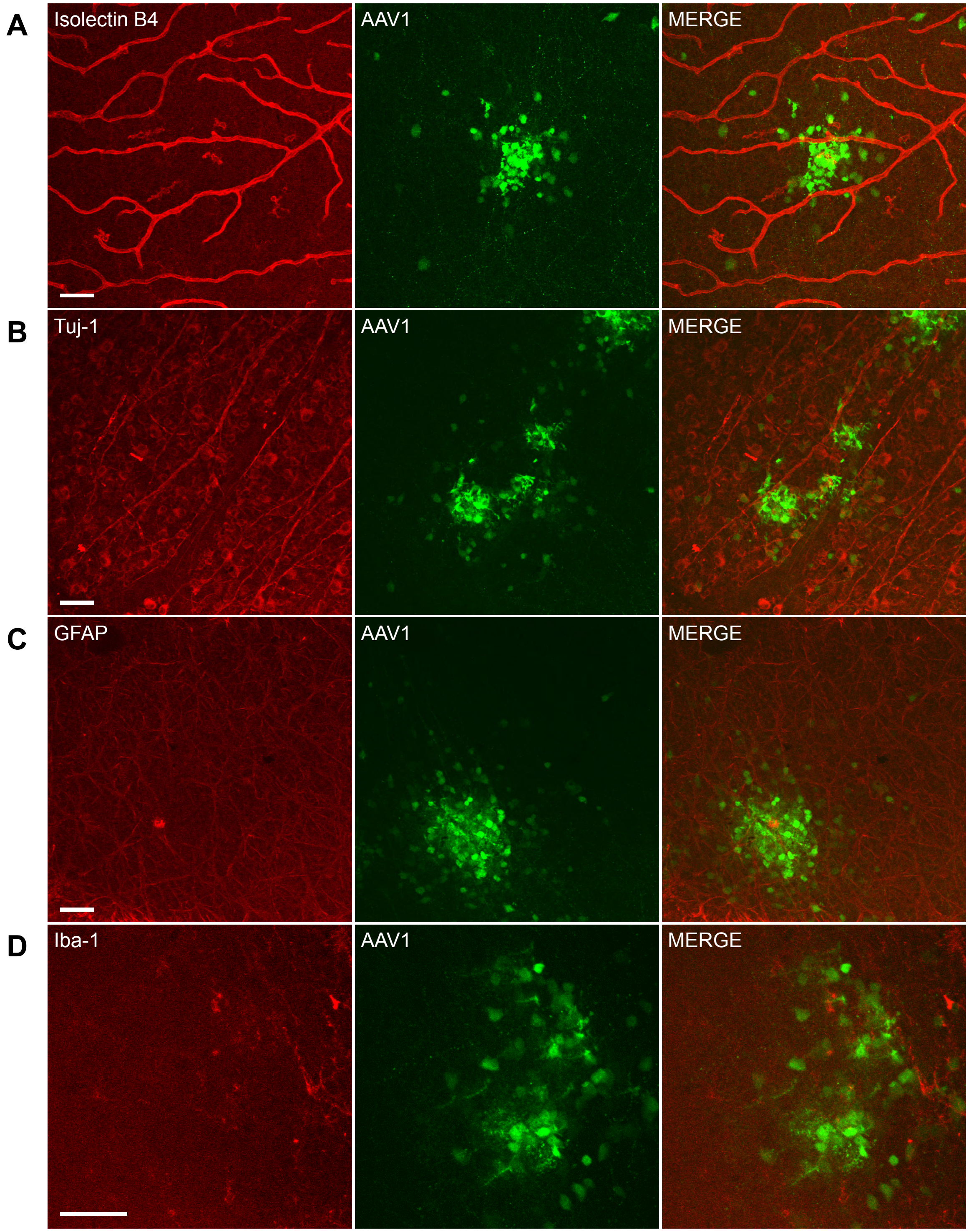

Supplemental Figure 1. Transduction characteristics of AAV1-GFP in the retina. Mice received intravitreal injection of AAV1-GFP (1 µl, $1x10^{12}$ vector genomes/ml) in both eyes. 8 weeks after injection, retinas were collected and stained with Isolectin B4 (A, red, vessels), anti-Tuj-1 (B, red, RGCs), anti-GFAP (C, red, astrocytes) and anti-Iba-1 (D, red, microglia/ monocytes). Confocal images were captured using 20X (A, B and C) or 40X (D) objective. Scale bar=50 µm.

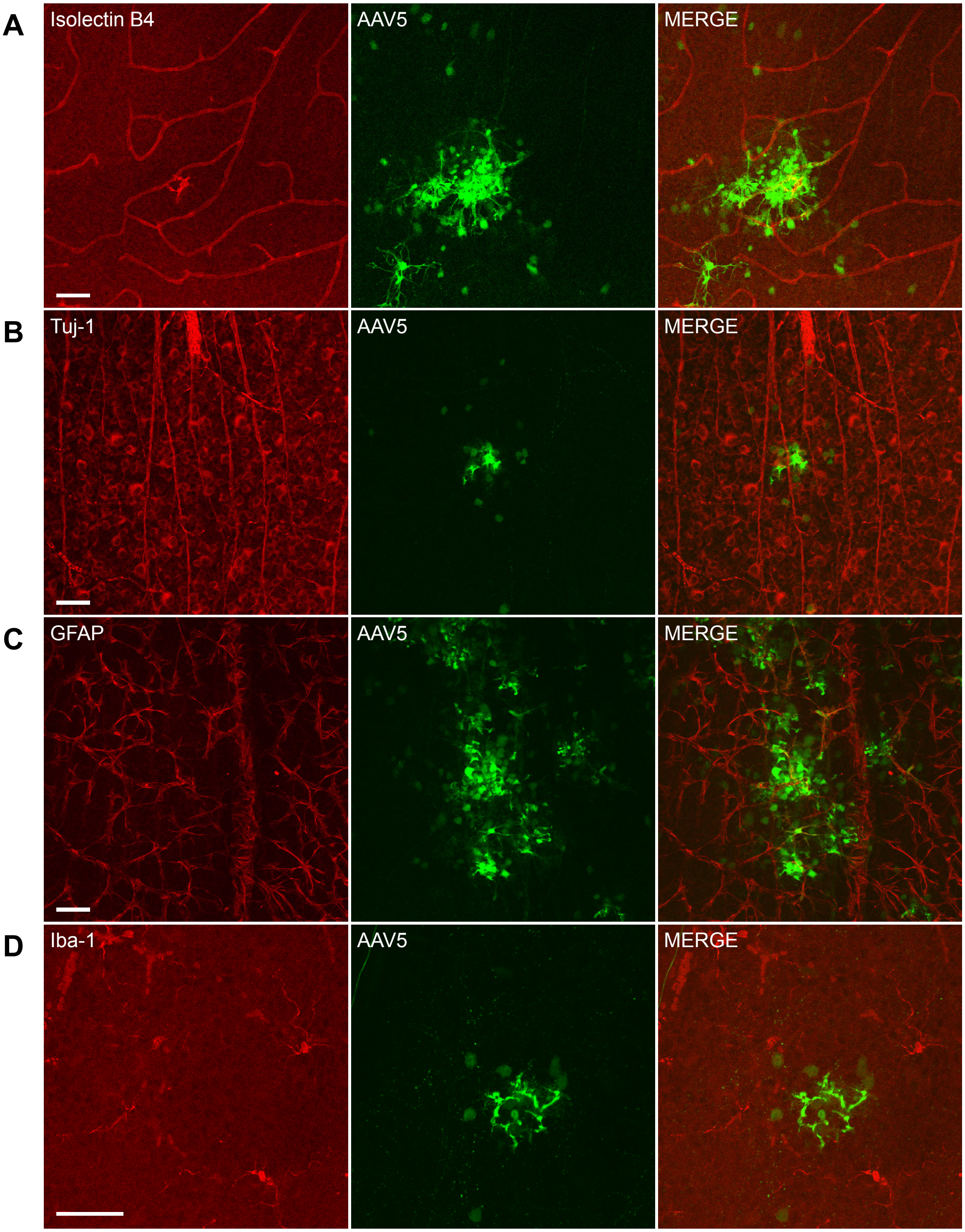

Supplemental Figure 2. Transduction characteristics of AAV5-GFP in the retina. Mice received intravitreal injection of AAV5-GFP (1 µl, 1×10^{12} vector genomes/ml) in both eyes. 8 weeks after injection, retinas were collected and stained with Isolectin B4 (A, red, vessels), anti-Tuj-1 (B, red, RGCs), anti-GFAP (C, red, astrocytes) and anti-Iba-1 (D, red, microglia/ monocytes). Confocal images were captured using 20X (A, B and C) or 40X (D) objective. Scale bar=50 µm.

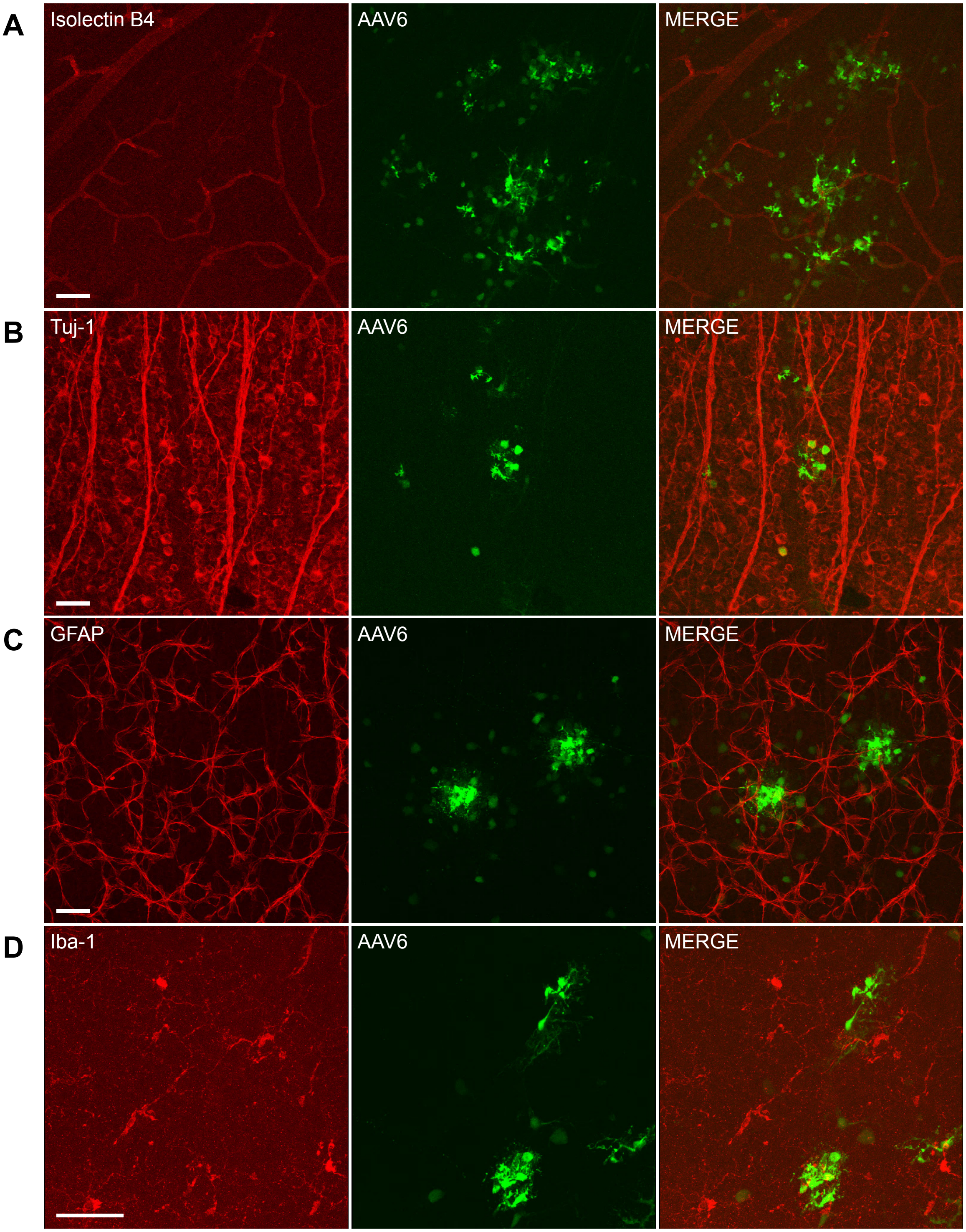

Supplemental Figure 3. Transduction characteristics of AAV6-GFP in the retina. Mice received intravitreal injection of AAV6-GFP (1 µl, 1×10^{12} vector genomes/ml) in both eyes. 8 weeks after injection, retinas were collected and stained with Isolectin B4 (A, red, vessels), anti-Tuj-1 (B, red, RGCs), anti-GFAP (C, red, astrocytes) and anti-Iba-1 (D, red, microglia/ monocytes). Confocal images were captured using 20X (A, B and C) or 40X (D) objective. Scale bar=50 µm.

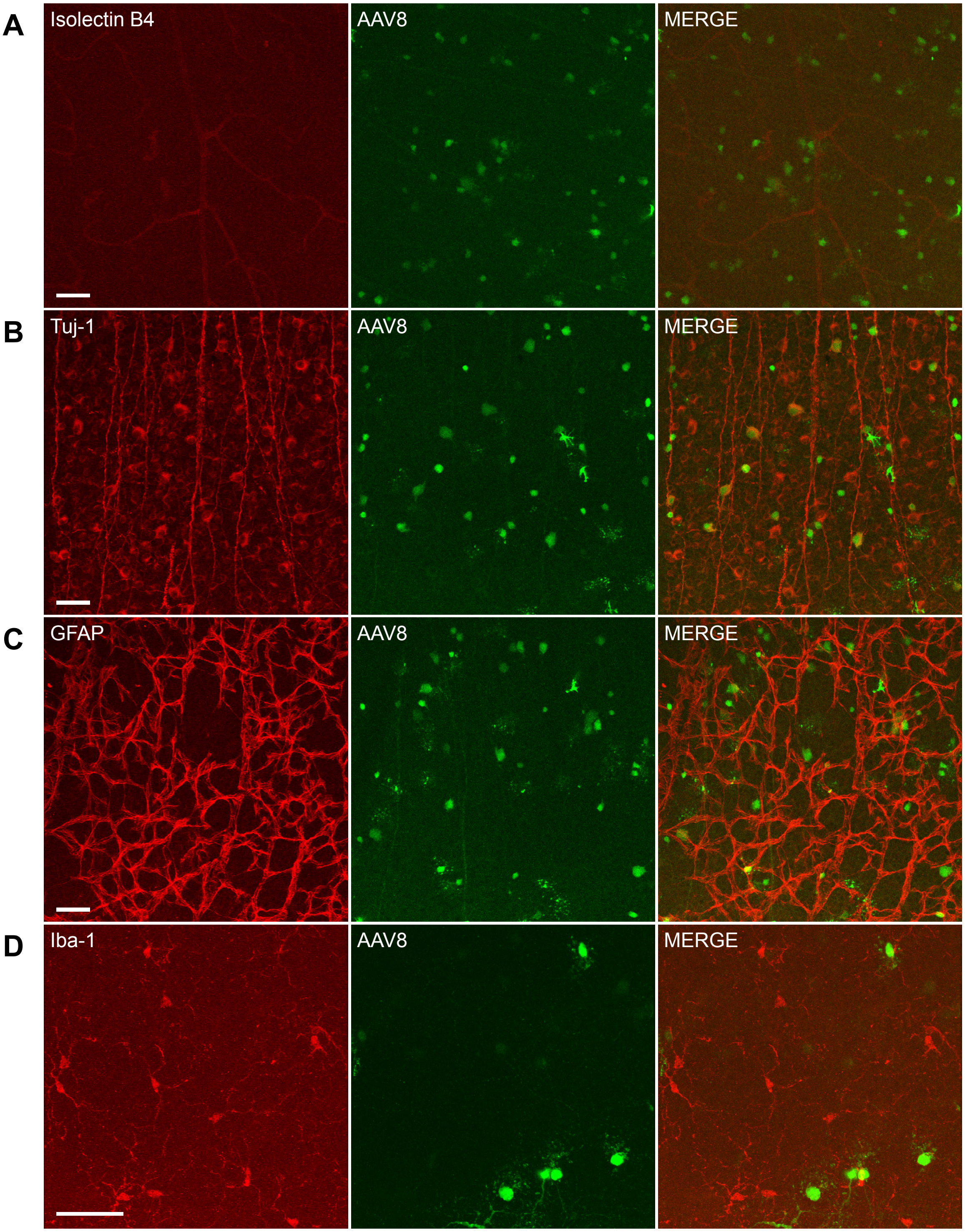

Supplemental Figure 4. Transduction characteristics of AAV8-GFP in the retina. Mice received intravitreal injection of AAV8-GFP (1 µl, $1x10^{12}$ vector genomes/ml) in both eyes. 8 weeks after injection, retinas were collected and stained with Isolectin B4 (A, red, vessels), anti-Tuj-1 (B, red, RGCs), anti-GFAP (C, red, astrocytes) and anti-Iba-1 (D, red, microglia/ monocytes). Confocal images were captured using 20X (A, B and C) or 40X (D) objective. Scale bar=50 µm.

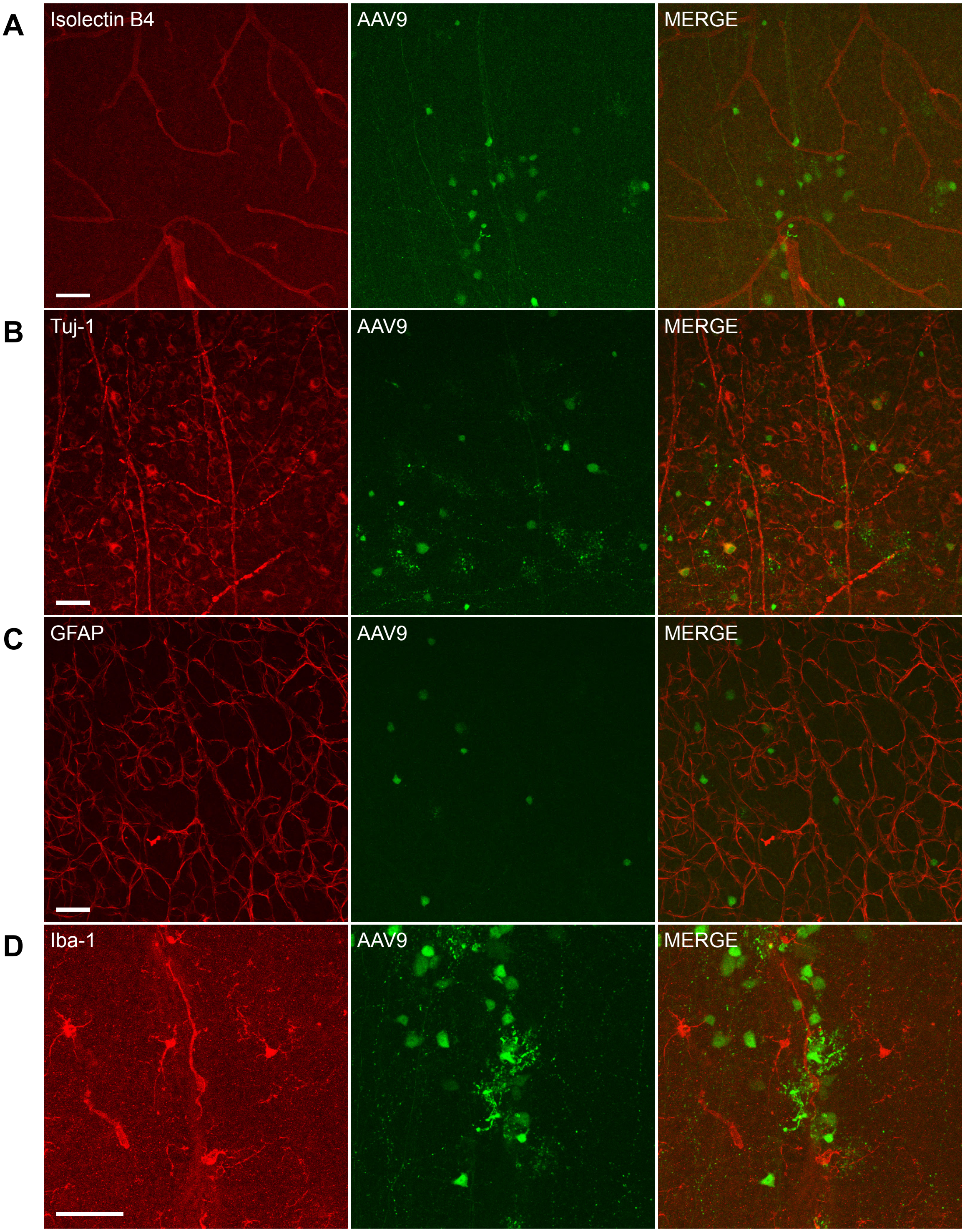

Supplemental Figure 5. Transduction characteristics of AAV9-GFP in the retina. Mice received intravitreal injection of AAV9-GFP (1 µl, $1x10^{12}$ vector genomes/ml) in both eyes. 8 weeks after injection, retinas were collected and stained with Isolectin B4 (A, red, vessels), anti-Tuj-1 (B, red, RGCs), anti-GFAP (C, red, astrocytes) and anti-Iba-1 (D, red, microglia/ monocytes). Confocal images were captured using 20X (A, B and C) or 40X (D) objective. Scale bar=50 µm.

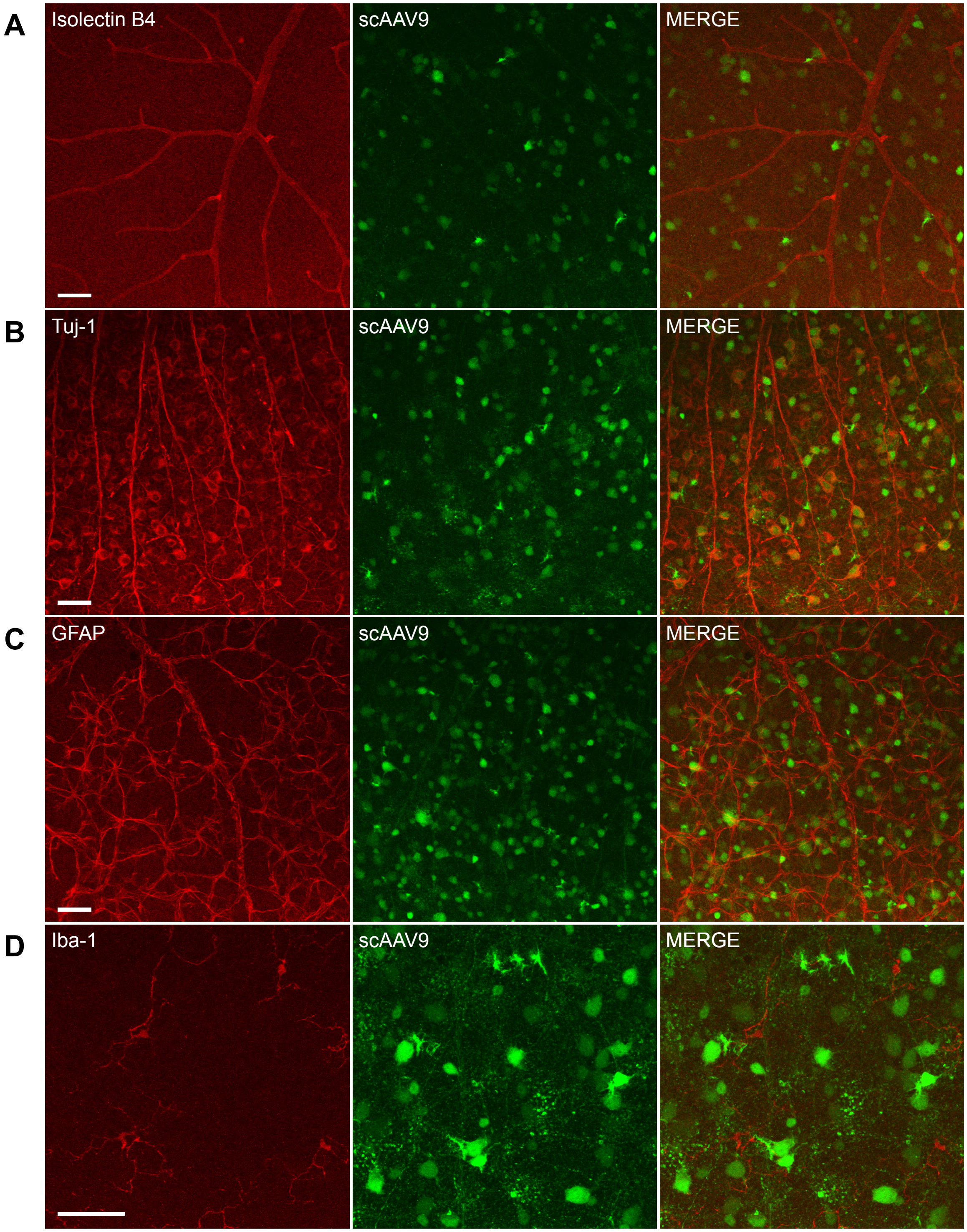

Supplemental Figure 6. Transduction characteristics of scAAV9-GFP in the retina. Mice received intravitreal injection of scAAV9-GFP (1 µl, $1x10^{12}$ vector genomes/ml) in both eyes. 8 weeks after injection, retinas were collected and stained with Isolectin B4 (A, red, vessels), anti-Tuj-1 (B, red, RGCs), anti-GFAP (C, red, astrocytes) and anti-Iba-1 (D, red, microglia/ monocytes). Confocal images were captured using 20X (A, B and C) or 40X (D) objective. Scale bar=50 µm.

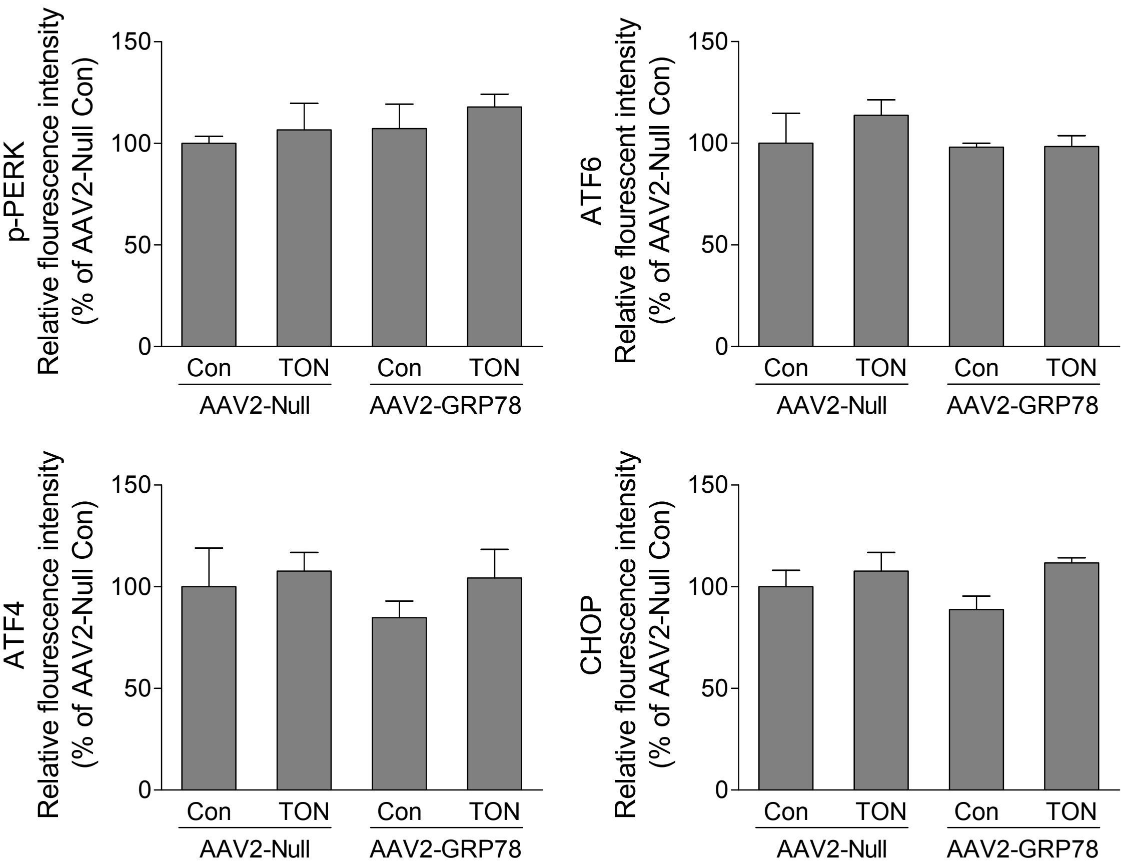

Supplemental Figure 7. The immunoreactivities of ER stress markers are not changed in the INL. TON was performed at 4-5 weeks after intravitreal injection of AAV2-Null or GRP78 and eyes were collected at 3 days after TON. Immunostaining for p-PERK, ATF6, ATF4 and CHOP expressions was performed on retinal frozen sections. Bar graphs represent the relative fluorescence intensities of p-PERK, ATF6, ATF4 and CHOP in the INL. INL: inner nuclear layer. n=3-4.

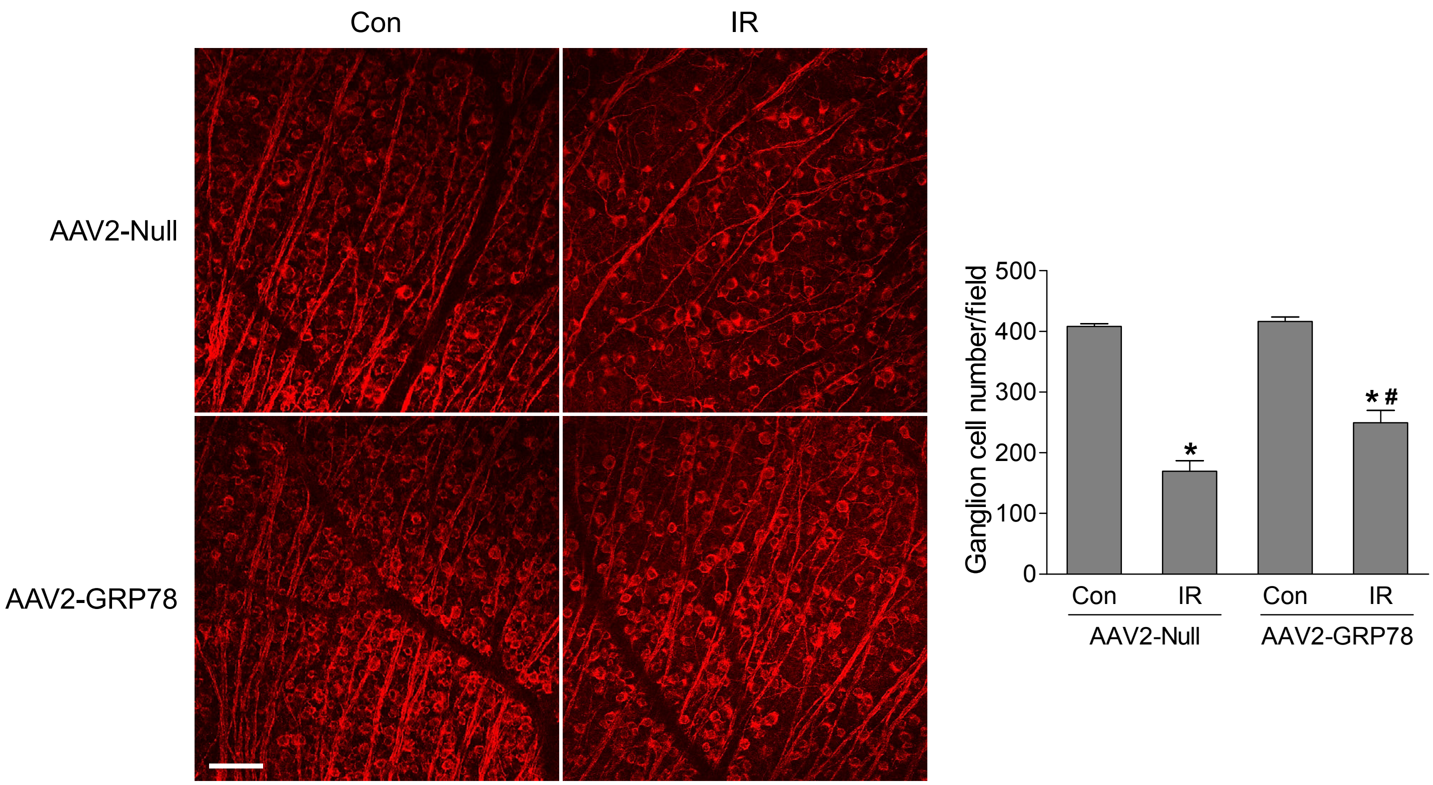

Supplemental Figure 8. **GRP78 overexpression attenuates RGC death after retinal ischemia-reperfusion (IR)**. IR was performed at 4-5 weeks after intravitreal injection of AAV2-Null or GRP78 and retinas were collected at 7 days after IR. Representative images of retinal flat-mounts labeled with Tuj-1 antibody (red) are shown. Bar graph represents the number of Tuj-1-positive cells per field. n=5-6, $^{*}p<0.05$ versus respective control, $^{\#}p<0.05$ AAV2-Null-IR versus AAV2-GRP78-IR. Scale bar=50 μm.

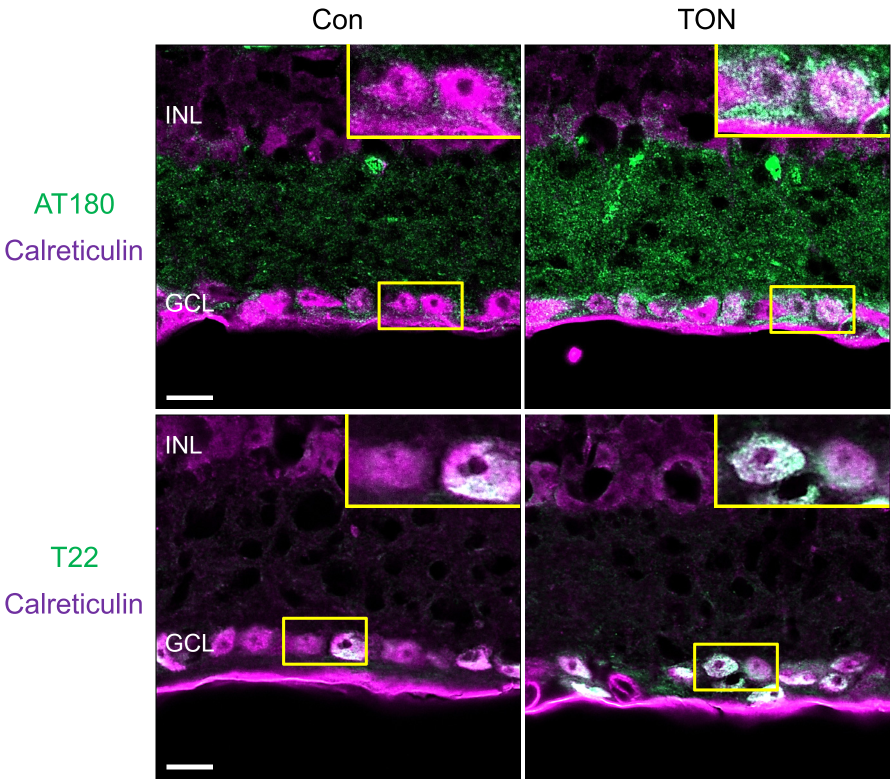

Supplemental Figure 9. Localization of phosphorylated tau and tau oligomers after TON. Eyes were collected at 3 days after TON and frozen retinal sections were immunostained with antibodies against phosphorylated tau (AT180) (green), tau oligomer (T22) (green) and ER marker (Calreticulin) (purple). Representative images were taken by confocal microscopy, with the insert showing a zoomed view of RGCs. GCL: ganglion cell layer; INL: inner nuclear layer. n=3-4. Scale bar=10 μm.

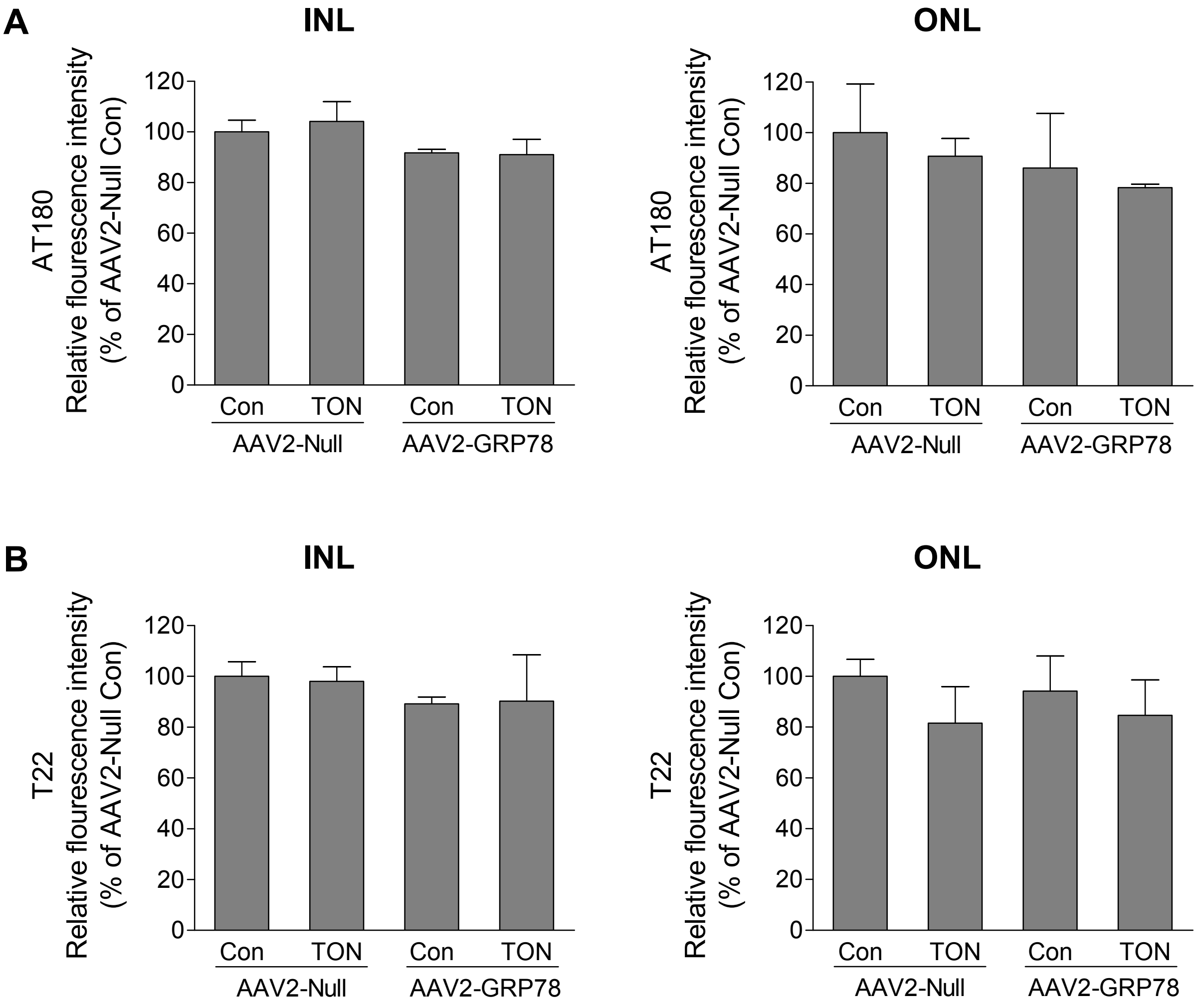

Supplemental Figure 10. **The immunoreactivities of phosphorylated tau and tau oligomer are not changed in the INL and ONL**. At 4-5 weeks after intravitreal injection of AAV2-Null or AAV2-GRP78, mice were subjected to TON. Eyes were collected 3 days after TON and retinal frozen sections were immunolabeled with antibodies against phosphorylated tau and tau oligomer. Bar graphs represent quantification of immunoreactivity of phosphorylated tau (A) and tau oligomer (B) in the INL and ONL. INL: inner nuclear layer; ONL: outer nuclear layer. n=3-4.

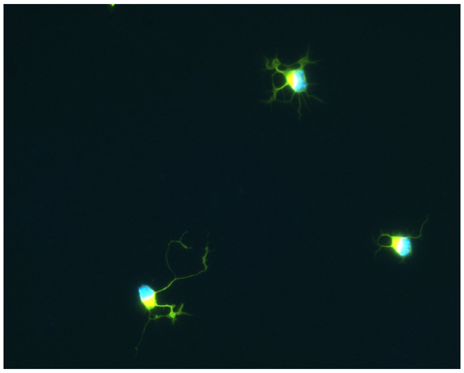

Supplemental Figure 11. **Validation of purity of isolated primary retinal ganglion cells (RGCs).** RGCs were isolated from mice pups at postnatal day 4-5 and confirmed with retinal ganglion cell marker Tuj-1 antibody (green). DAPI (blue) stains nuclei.

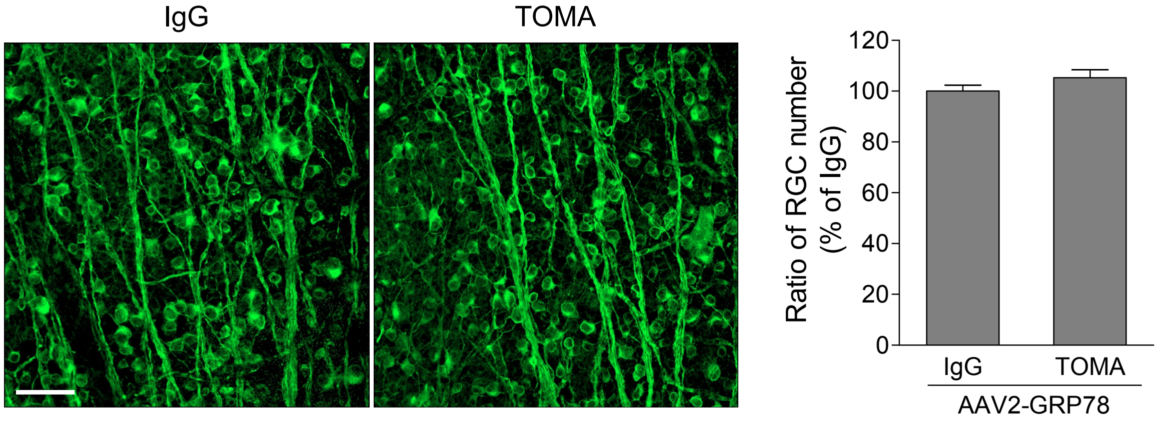

Supplemental Figure 12. **TOMA does not alter GRP78-induced RGC preservation after TON.** AAV2-GRP78 delivered WT mice were intravenously injected with vehicle (IgG) or TOMA (30 μg/mouse) 1 hour before TON. Retinas were collected at 7 days after TON. Representative images of retinal flat-mounts labeled with anti-Tuj-1 antibody (green) are shown. Bar graph represents the relative number of Tuj-1-positive cells per field. n=4-5. Scale bar=50 μm.
